# Real-Life Data on Readmissions of Worsening Heart Failure Outpatients in a Heart Failure Clinic

**DOI:** 10.7759/cureus.35611

**Published:** 2023-02-28

**Authors:** Hugo Inácio, Anabela De Carvalho, Joana Gamelas De Carvalho, André Maia, Gonçalo Durão-Carvalho, Joana Duarte, Catarina Rodrigues, Inês Araújo, Célia Henriques, Candida Fonseca

**Affiliations:** 1 Internal Medicine, Centro Hospitalar Universitário Lisboa Central - Hospital de Santo António dos Capuchos, Lisbon, PRT; 2 Internal Medicine, Hospital Senhora da Oliveira, Guimarães, PRT; 3 Internal Medicine, Hospital Amato Lusitano, Castelo Branco, PRT; 4 Internal Medicine, Centro Hospitalar de Trás-os-Montes e Alto Douro - Hospital de Vila Real, Vila Real, PRT; 5 Internal Medicine, Centro Hospitalar do Oeste - Unidade de Caldas da Rainha, Caldas da Rainha, PRT; 6 Intermediate Medical Care Unit, Internal Medicine, Centro Hospitalar de Lisboa Ocidental - Hospital de São Francisco Xavier, Lisbon, PRT; 7 Heart Failure Clinic, Internal Medicine, Centro Hospitalar de Lisboa Ocidental - Hospital de São Francisco Xavier, Lisbon, PRT; 8 Centro de Estudos de Doenças Crónicas (CEDOC), NOVA Medical School - Faculdade de Ciências Médicas, Universidade Nova de Lisboa, Lisbon, PRT

**Keywords:** mortality, heart failure management program, predictors of readmission, readmission, worsening heart failure, heart failure

## Abstract

Introduction

Recurrent hospitalizations for worsening heart failure (WHF) represent a major global public health concern, resulting in significant individual morbimortality and socioeconomic costs. This real-life study aimed to determine the rate and predictors of readmission for WHF in a cohort of outpatients with chronic heart failure (CHF) followed in a heart failure clinic (HFC) at a university hospital.

Methods

We conducted a longitudinal, observational, and retrospective study of all consecutive CHF patients seen at the HFC of the São Francisco Xavier Hospital, Lisbon, by a multidisciplinary team in 2019. The patients were followed for one year and were on optimized therapy. The inclusion criteria for the study were patients who had been hospitalized and subsequently discharged at least three months prior to their enrollment. Patient demographics, heart failure (HF) characterization, comorbidities, pharmacological treatment, treatments of decompensated HF in the day hospital (DH), hospitalizations for WHF, and death were recorded. We applied logistic regression analysis to assess predictors of hospital readmission for HF.

Results

A total of 351 patients were included: 90 patients (26%) had WHF requiring treatment with intravenous diuretics in the DH; 45 patients (mean age: 79.1 ± 9.0 years) were readmitted for decompensated HF within one year (12.8%) with no gender difference, while 87.2% of the patients (mean age: 74.9 ± 12.1 years) were never readmitted. Readmitted patients were significantly older than those who were not (p=0.031). Additionally, they had a higher New York Heart Association (NYHA) functional classification (p<.001), were on a higher daily dose of furosemide (p=0.008) at the time of the inclusion visit, were more frequently affected by the chronic obstructive pulmonary disease (COPD) (p=0.004); had been treated more often in the DH for WHF (p<.001) and had a higher mortality rate (p<.001) at one year.

Conclusions

This study aimed to determine WHF patient readmission rates and predictors. According to our results, a higher NYHA class, the need for treatment in the DH for WHF, a daily dose of furosemide equal to or greater than 80 mg, and COPD were predictors of readmission for WHF. CHF patients continue to experience WHF and recurrent hospitalizations despite therapeutic advances and close follow-up in the HFC with the multidisciplinary team. Besides COPD, the HF readmission risk factors found were mainly related to advanced disease. Furthermore, the structured and multidisciplinary approach of our disease management program likely contributed to our relatively low rate of readmissions.

## Introduction

Heart failure (HF) currently represents a global epidemic with a 1-2% prevalence in high-income countries and can reach 11.8% of the population aged 65 or older [1]. In Portugal, the estimated prevalence of heart failure was 5.2% in 2018 (according to the Epidemiology of Heart Failure and Learning study) and is projected to increase by 30% by 2035 and 33% by 2060, due to increased longevity and aging of the population [2,3].

The economic burden of HF on worldwide healthcare systems and economies is substantial and is likely to increase due to the disease's growing prevalence [4]. The total costs attributable to HF in Portugal were approximately € 405 million in 2014, accounting for approximately 2.6% of healthcare expenses among Portugal’s 10 million inhabitants. [5]. The projection of total annual costs of HF up to 2036 suggests a 24% increase between 2014 (€ 405 million) and 2036 (€ 503 million), with hospitalizations representing most of these costs [5]. Despite therapeutic advances in recent years, HF remains the leading cause of hospital admission among older patients, with high morbidity and mortality [6,7].

The clinical course of most patients with HF is characterized by periods of clinical stability and episodes of worsening symptoms [8,9]. Worsening HF (WHF) is defined by the development of progressively increasing symptoms and signs of HF requiring intravenous (IV) diuretic treatment [8,9]. WHF should be differentiated from the term acute HF (AHF) as it requires a diagnosis of chronic HF (CHF) but has an equally poor prognosis [8-10]. While AHF leads to hospitalization, patients can experience WHF in the outpatient setting. WHF patients can avoid hospitalization or emergency department (ED) care by adjusting their therapy appropriately [8]. Hospitalization for WHF significantly impacts patients’ quality of life since each new event leads to a functional decline [8,11] and represents the main prognostic factor for increased mortality [6].

HF patients often have comorbidities that add to clinical complexity, increasing the disease management’s difficulty and worsening outcomes [11,12]. Data from the European Society of Cardiology (ESC) Heart Failure Pilot Survey indicate that 74% of HF patients have at least one comorbidity [13]. These diseases represent risk factors for cardiac decompensation, and their control is essential to avoid WHF and hospitalizations. This study aimed to evaluate the clinical and demographic characteristics of a cohort of CHF outpatients in an HF clinic (HFC) and determine the predictors of their WHF-related readmission rate at one year.

## Materials and methods

We conducted a retrospective, observational, longitudinal study of all consecutive outpatients with CHF. Following their first hospitalisation, these patients were referred to the HFC at São Francisco Xavier Hospital, which is part of Centro Hospitalar de Lisboa Ocidental, a tertiary hospital in Portugal. The patients were followed for one year and included in the study if they were on optimized therapy and had not been readmitted in the three months before their inclusion visit. We used the HFC database to identify the patients and collected demographic and clinical data from the patients’ electronic medical records. All patients whose information was incomplete in the medical record at the visit were excluded from the cohort.

Variables

Data collection included sociodemographic information, HF characteristics, comorbid conditions, pharmacological therapy, WHF treatments in the day hospital (DH), hospitalizations for WHF, and death at one year. We recorded patients’ HF etiology as either ischemic or nonischemic as noted in their medical records along with their left ventricular ejection fraction (LVEF) phenotype and the New York Heart Association (NYHA) functional classification. The LVEF was classified as preserved, or reduced, while the NYHA functional classification ranged from I to IV. The comorbidities included arterial hypertension, atrial fibrillation, type two diabetes, chronic kidney disease, chronic obstructive pulmonary disease (COPD), obesity, obstructive sleep apnea syndrome, and tobacco and alcohol consumption. The study’s primary endpoint was hospitalization for WHF, and all-cause mortality was analyzed as a secondary endpoint.

Statistical analysis

Continuous variables are presented with the mean and standard deviation and categorical variables are presented as absolute and relative frequencies. The differences between hospitalized and non-hospitalized patients were calculated using the chi-square test for categorical variables. In the case of continuous variables, such as age and furosemide daily dose, we used the Mann-Whitney test after the Shapiro-Wilk test rejected their normality.

A logistic regression model was constructed to assess predictors of HF hospitalization one year after the inclusion visit. The variables considered optimal for inclusion in the logistic regression model were those that satisfied the criterion of having an unadjusted association with hospital admission for decompensated HF and a p-value <0.05. Under these criteria, the following variables were selected: NYHA classification, treatment in the DH for WHF, COPD, and a daily dose of furosemide ≥80 mg. Age and gender were included as independent variables. The discrimination of the models was estimated by constructing a receiver operator characteristic curve (ROC) and measuring the area under the curve (AUC). Data analysis was performed using JASP® (version 0.16.4; JASP Team, University of Amsterdam, The Netherlands) statistical software.

## Results

Of the 418 screened patients, 67 (16%) were excluded for incomplete medical records. Therefore, 351 patients were analyzed in the study. Table 1 describes the sample characteristics and the analysis between the two study groups: patients hospitalized for WHF during follow-up and those who were not.

**Table 1:** Baseline characteristics grouped by WHF readmission status for one-year follow-up Day hospital, patients treated in day hospital for worsening heart failure symptoms. Day hospital > 1, more than a day of treatment in day hospital. *Five patients were removed because early treatment of decompensation was not the reason for observation. ACEIs: angiotensin-converting enzyme inhibitors; ARBs: angiotensin receptor blockers; ARNI: angiotensin receptor/neprilysin inhibitor; BB: beta blocker; CKD: chronic kidney disease; COPD: chronic obstructive pulmonary disease; HF: heart failure; HF: heart failure; HFrEF: heart failure with reduced ejection fraction (<50%); HFpEF: heart failure with preserved ejection fraction (≥ 50%); MRA: mineral corticoid antagonist; NYHA: New York Heart Association; OSAS: obstructive sleep apnea syndrome; SGLT2i: sodium–glucose cotransporter 2 (SGLT2) inhibitors; WHF: worsening heart failure.

Variables	Overall	Admitted	Not admitted	P-value
	351 (100%)	45 (12.8%)	306 (87.2%)	
Demographics				
Female, n (%)	150 (42.7%)	24 (53.3%)	126 (41.2%)	0.124
Male, n (%)	201 (57.3%)	21 (46.7%)	180 (58.8%)	
Age in years, mean (SD)	75.4 (11.8)	79.1 (9.0)	74.9 (12.1)	0.031
HF evaluation				
HF type, n (%)				
HFrEF	114 (32.5%)	17 (37.8%)	97 (31.7%)	0.416
HFpEF	237 (67.5%)	28 (62.2%)	209 (68.3%)	0.292
NYHA, median	2.0	2.5	2.0	<0.001
Etiology, n (%)				
Ischemic	103 (29.3%)	12 (26.7%)	91 (29.7%)	0.673
Nonischemic	248 (70.6%)	33 (73.3%)	215 (70.3 %)	0.535
Comorbidities, n (%)				
Hypertension	186 (53.0%)	25 (55.6%)	161 (52.6%)	0.712
Diabetes	215 (61.2%)	31 (68.9%)	184 (60.1%)	0.260
Atrial fibrillation	159 (45.3%)	21 (46.7%)	138 (45.1%)	0.844
COPD	171 (48.7%)	31 (68.9%)	140 (45.8%)	0.004
CKD	158 (45.0%)	20 (44.4%)	138 (45.1%)	0.934
Obesity	198 (56.4%)	21 (46.7%)	177 (57.8%)	0.158
OSAS	185 (52.7%)	23 (51.1%)	162 (52.9%)	0.818
Tobacco use	177 (50.4%)	17 (37.8%)	160 (52.3%)	0.175
Comorbidities number, mean (SD)	4.1 (1.4)	4.1 (1.4)	4.1 (1.3)	0.301
Medications, n (%)				
ACEIs	109 (31.0%)	15 (33.3%)	94 (30.7%)	0.723
ARBs	91 (25.9%)	11 (24.4%)	80 (26.1%)	0.808
ARNI	111 (31.6%)	12 (26.7%)	99 (32.4%)	0.444
BBs	162 (46.2%)	25 (55.6%)	137 (44.8%)	0.359
MRAs	171 (48.7%)	16 (35.6%)	155 (50.6%)	0.058
SGLT2i	196 (55.8%)	23 (51.1%)	173 (56.5%)	0.494
Furosemide mean dose (SD)	55.8 (41.3)	76.0 (46.4)	52.8 (39.7)	0.008
Furosemide ≤ 40 mg id	189 (53.8%)	14 (31.1%)	175 (57.2%)	0.001
Furosemide ≥ 80 mg id	108 (30.8%)	25 (55.6%)	83 (27.1%)	<0.001
Metolazone	190 (54.1%)	24 (53.3%)	166 (54.2%)	0.908
Follow up				
Day hospital, n (%)	90* (26.0%)	21 (46.7%)	69 (22.5%)	<0.001
Day hospital > 1	42 (12.1%)	14 (31.1%)	28 (9.1%)	<0.001
Mortality, n (%)	43 (12.2%)	15 (33.3%)	28 (9.1%)	<0.001

Of the 351 included patients, the mean age was 74.5 years old (± 11.8 years), 42.7% were women, and 57.3% were men. At the one-year follow-up, 45 patients (12.8%) had at least one WHF hospitalization and were older (p=.0031) than those who had not been hospitalized. No statistically significant difference was found related to patient sex (p=0.124). We also found no remarkable differences in HF type and aetiology (p>0.05). However, we found a statistically significant difference regarding baseline NYHA functional classification: patients who were hospitalized during follow-up had, at the inclusion visit, a median NYHA classification of 2.5, which was higher than those who were not hospitalized (median of 2; p<.001).

At the one-year follow-up, 90 patients (26%) received IV furosemide at DH for WHF symptoms, and 42 patients (46.7%) required more than one day of treatment. Of these 90 patients, 69 (76.7%) were not hospitalized. Both groups had a mean of 4.1 comorbidities. Only COPD showed a higher prevalence among hospitalized patients (68.9%) versus non-hospitalized patients (45.8%; p=0.004). Hospitalized patients had a significantly higher chronic daily dose of furosemide (p=0.008) than non-hospitalized patients. Furthermore, more than 50% of the hospitalized patients took a dose of furosemide ≥ 80 mg (p<.001). Patients hospitalized for WHF had a 33.3% mortality rate during follow-up, significantly higher than those who were not (9.1%; p<.001).

Table 2 presents the results of a logistic regression model (ROC curve with AUC, 0.793) to determine the independent predictors of hospitalization within one year after the inclusion visit. Compared to non-hospitalized patients, the patients who were hospitalized were more likely to have a higher NYHA class (odds ratio (OR), 2.622; p=0.004), treated at least once in the DH for WHF (OR, 3.289; p=0.002) and on a chronic daily dose of furosemide of ≥ 80 mg (OR, 2.421; p=0.019). COPD was significantly associated with a higher risk of readmission (OR, 2.878; p=0.007).

**Table 2:** Result of the logistic regression model on the independent predictors of readmission for WHF Day hospital, patients treated in a day hospital for worsening heart failure symptoms. COPD: chronic obstructive pulmonary disease; CI: confidence interval; NYHA: New York Heart Association; WHF: worsening heart failure.

Variable	Odds Ratio (95% CI)	P-value
Age	1.022 (0.987-1.059)	0.225
Sex	1.061 (0.494-2.278)	0.880
NYHA	2.622 (1.349-5.098)	0.004
Day hospital	3.289 (1.5716.886)	0.002
Furosemide ≥ 80 mg id	2.421 (1.155-5.074)	0.019
COPD	2.878 (1.337-6.198)	0.007

## Discussion

Despite advances in managing HF in recent decades, it remains one of the leading causes of hospitalization in patients aged 65 years or older, especially for those over 80 [14]. WHF and repeated hospitalizations represent the main factor of poor prognosis and have high individual and socioeconomic costs. Reducing rehospitalization remains one of the main goals of the São Francisco Xavier Hospital HFC, which offers a multidisciplinary integrated HF management program and centralizes and optimizes care for patients with HF strictly following the diagnostic and treatment protocols based on the ESC guidelines.

Our results showed an aging population with a higher percentage of women and HF with preserved LVEF. The readmission rate for WHF was 12.8%. In our 351-patient cohort, one in four had WHF symptoms requiring IV diuretic treatment in the DH. WHF patients who were hospitalized were typically in an advanced stage of the disease and had higher basal NYHA class, a higher daily dose of furosemide, and a higher rate of DH treatment for worsening symptoms. COPD was also associated with a higher risk of readmission. The mortality rate for all causes was 12.2%.

Clinical trials and registries exclusively from cardiology departments are usually not representatives of the general population, as they tend to include highly selected patients who are younger, with a clear prevalence of males and reduced ventricular function [15]. For this reason, real-life registries and studies like ours continue to be necessary to provide real-world data on HF patients [15,16]. Compared to a large-scale registry, such as the EURObservational programme, conducted in 136 Cardiology Centres, where the mean age in the CHF group was 66 years [17], our study population was older, with a mean age of 75.4 years (± 11.8 years), and 42.7% of the participants were women compared to 29.7% in the EURObservational programme [17]. In addition, only 29.3% of our patients had HF of ischemic origin, in contrast to 40.5% in the EURObservational programme [17]. The higher mean age and proportion of women in our cohort are in line with our higher prevalence of HF with preserved ejection fraction (HFpEF), which is more frequently caused by nonischemic heart disease [18], namely arterial hypertension.

In our study, 12.8% of patients were readmitted for WHF during the one-year follow-up period. The literature reports higher rates of hospitalization; recent studies from Sweden, Spain, and Japan reported 38.4%, 44.7%, and 27.2% readmission rates, respectively, for patients in usual care after discharge from decompensated HF [15,18,19]. According to the study conducted in Spain that examined the population over the first two decades of the 21st century, the number of HF readmissions has increased significantly over the last five years [15]. Aging, comorbidities, and inefficient follow-up are probable reasons for this increase [15].

In addition, our study’s lower rate of hospitalization may be attributed to an efficient follow-up program in our clinic, which is structured and performed by a multidisciplinary team [20]. The following factors contributed to this: (1) patients with CHF are managed by the clinic’s multidisciplinary team per protocols established by the ESC, including the optimization of prognostic-modifying therapies and control of comorbidities; (2) both patients and caregivers are educated about symptoms of WHF, particularly congestion-weight gain, edema, and dyspnea and are encouraged to contact the team; and (3) when there is a need for contact, a telephone assessment is conducted, and if necessary, patients are observed in the DH for diagnosis and treatment. As a result, most patients with early HF decompensation do not require hospitalization and are treated in an outpatient setting. Similar results have been found in other studies in this area, in which disease management programs were compared with usual care, and the former significantly reduced hospitalizations [7,21-25].

Nevertheless, the higher hospital care of patients to the DH with WHF symptoms reveals that all patients with CHF are potential WHF patients, and new therapies that reduce the frequency of WHF are needed. In 2020, the Vericiguat Global Study in Subjects with Heart Failure with Reduced Ejection Fraction (VICTORIA) study showed that vericiguat decreased the incidence of death from cardiovascular causes or hospitalization for HF among patients with high-risk HF [26]. However, until its use is widespread or new molecules are discovered, the best strategy seems to be to refer patients to integrated HF management programs.

The predictors of hospitalization found in the logistic regression model were NYHA class, treatment in HD for WHF, chronic daily dose of furosemide of 80 mg or higher, and COPD. NYHA functional classification is a significant independent predictor of HF prognosis [2]. Further, congestion has been identified as the leading cause of hospitalization in HF [6], and those patients need a higher daily dose of diuretic therapy. Our center uses successively higher doses of furosemide, and, when necessary, we combine furosemide with low-dose metolazone until euvolemia is achieved. We found no statistically significant difference between hospitalized and nonhospitalized patients in terms of metolazone use. Furthermore, the fact that many patients require both medications suggests a more difficulty in managing congestion.

These factors reflect the severity of our patients’ disease and show that those in a more advanced stage were most frequently hospitalized for WHF. Our disease management program effectively prevented WHF hospitalization in severe HF patients even when decompensated, highlighting the importance of early management and structured follow-up of WHF to prevent functional decline.

COPD is estimated to affect approximately 20% of HF patients and can significantly impact symptom management and outcome [12]. In other studies, COPD was also identified as a risk factor for hospitalization for decompensated HF [6,13,15,27,28]. The small size of the readmitted group could have been insufficient to detect statistically significant associations with the other comorbidities.

Long-term all-cause mortality rate of the total cohort during follow-up was 12.2%, with a mortality of 33.3% of those rehospitalized vs. 9.1% (p<.001) of the non-readmitted group. This mortality is slightly higher than that reported by the EURObservational programme for chronically ill patients [17] but lower than that reported in other more recent real-life studies, which was higher than 20% [15,18].

The present study had several limitations. The effect of follow-up at the HFC on reducing hospitalizations and mortality was not compared with a control group within the same population. The small sample size of hospitalized patients for WHF did not permit the identification of any other statistically significant relationship with other comorbidities. Also, socioeconomic status, cognitive frailty, and other known worse prognosis factors should have been included as variables. A future study that includes a cost-effectiveness analysis of the integrated disease management program would provide important information on the economic benefits of this approach for patients.

## Conclusions

Despite therapeutic advances and HF management programs, patients with CHF have high rates of WHF and recurrent hospitalizations. Our study showed that even with optimized therapy, HF patients will present with WHF requiring intravenous diuretic therapy. Furthermore, despite the limitations mentioned, those who required hospitalization were those with a higher NYHA class, were more often treated in DH for WHF, and required a higher daily dose of diuretic therapy. These, together with COPD, were the predictors of readmission in our study. These data suggest that integrated follow-up of these patients has a positive effect on reducing hospitalizations as early decompensation is rapidly recognized and treated. On the other hand, there is a group of patients who, even with optimized therapy, will require hospitalization due to more advanced disease. Until novel molecules are available to reduce WHF, patients can benefit from integrated disease management programs. These programs seem to effectively improve patients’ quality of life by avoiding rehospitalization and reducing morbidity and the socioeconomic burden of the disease.

## References

[1] Groenewegen A, Rutten FH, Mosterd A, Hoes AW (2020). Epidemiology of heart failure. Eur J Heart Fail.

[2] Fonseca C, Brás D, Araújo I, Ceia F (2018). Heart failure in numbers: estimates for the 21st century in Portugal. Rev Port Cardiol (Engl Ed).

[3] Gouveia M, Ascenção R, Fiorentino F (2019). The current and future burden of heart failure in Portugal. ESC Heart Fail.

[4] Savarese G, Becher PM, Lund LH, Seferovic P, Rosano GM, Coats AJ (2023). Global burden of heart failure: a comprehensive and updated review of epidemiology. Cardiovasc Res.

[5] Gouveia MR, Ascenção RM, Fiorentino F, Costa JN, Broeiro-Gonçalves PM, Fonseca MC, Borges MF (2020). Current costs of heart failure in Portugal and expected increases due to population aging. Rev Port Cardiol (Engl Ed).

[6] Gheorghiade M, Vaduganathan M, Fonarow GC, Bonow RO (2013). Rehospitalization for heart failure: problems and perspectives. J Am Coll Cardiol.

[7] Atienza F, Anguita M, Martinez-Alzamora N (2004). Multicenter randomized trial of a comprehensive hospital discharge and outpatient heart failure management program. Eur J Heart Fail.

[8] Greene SJ, Mentz RJ, Felker GM (2018). Outpatient worsening heart failure as a target for therapy: a review. JAMA Cardiol.

[9] Butler J, Yang M, Manzi MA, Hess GP, Patel MJ, Rhodes T, Givertz MM (2019). Clinical course of patients with worsening heart failure with reduced ejection fraction. J Am Coll Cardiol.

[10] Davison BA, Senger S, Sama IE (2021). Is acute heart failure a distinctive disorder? An analysis from BIOSTAT-CHF. Eur J Heart Fail.

[11] Goldgrab D, Balakumaran K, Kim MJ, Tabtabai SR (2019). Updates in heart failure 30-day readmission prevention. Heart Fail Rev.

[12] McDonagh TA, Metra M, Adamo M (2021). 2021 ESC guidelines for the diagnosis and treatment of acute and chronic heart failure. Eur Heart J.

[13] Su A, Al'Aref SJ, Beecy AN, Min JK, Karas MG (2019). Clinical and socioeconomic predictors of heart failure readmissions: a review of contemporary literature. Mayo Clin Proc.

[14] Fudim M, O'Connor CM, Dunning A (2018). Aetiology, timing and clinical predictors of early vs. late readmission following index hospitalization for acute heart failure: insights from ASCEND-HF. Eur J Heart Fail.

[15] Fernández-Bergés D, González-Fernández R, Félix-Redondo FJ (2022). Clinical and prognostic profile evolution of patients discharged from hospital due to heart failure in the first two decades of the 21st century. The INCA-Ex Registry. Aten Primaria.

[16] Trullàs JC, Miró Ò, Formiga F (2016). The utility of heart failure registries: a descriptive and comparative study of two heart failure registries. Postgrad Med J.

[17] Maggioni AP, Dahlström U, Filippatos G (2013). EURObservational research programme: regional differences and 1-year follow-up results of the heart failure pilot survey (ESC-HF Pilot). Eur J Heart Fail.

[18] Wideqvist M, Cui X, Magnusson C, Schaufelberger M, Fu M (2021). Hospital readmissions of patients with heart failure from real world: timing and associated risk factors. ESC Heart Fail.

[19] Kato M, Mori Y, Watanabe D, Onoda H, Fujiyama K, Toda M, Kito K (2022). Discharge disposition and 1-year readmission in acute-phase hospitalized patients with heart failure: a retrospective observational multi-center study. Heart Vessels.

[20] Fonseca C (2018). A heart failure clinic. Eur Heart J.

[21] Capomolla S, Febo O, Ceresa M (2002). Cost/utility ratio in chronic heart failure: comparison between heart failure management program delivered by day-hospital and usual care. J Am Coll Cardiol.

[22] Kasper EK, Gerstenblith G, Hefter G (2002). A randomized trial of the efficacy of multidisciplinary care in heart failure outpatients at high risk of hospital readmission. J Am Coll Cardiol.

[23] Van Spall HGC, Rahman T, Mytton O (2017). Comparative effectiveness of transitional care services in patients discharged from the hospital with heart failure: a systematic review and network meta-analysis. Eur J Heart Fail.

[24] Feltner C, Jones CD, Cené CW (2014). Transitional care interventions to prevent readmissions for persons with heart failure: a systematic review and meta-analysis. Ann Intern Med.

[25] Blum MR, Øien H, Carmichael HL, Heidenreich P, Owens DK, Goldhaber-Fiebert JD (2020). Cost-effectiveness of transitional care services after hospitalization with heart failure. Ann Intern Med.

[26] Armstrong PW, Pieske B, Anstrom KJ (2020). Vericiguat in patients with heart failure and reduced ejection fraction. N Engl J Med.

[27] Mirkin KA, Enomoto LM, Caputo GM, Hollenbeak CS (2017). Risk factors for 30-day readmission in patients with congestive heart failure. Heart Lung.

[28] Arora S, Patel P, Lahewala S (2017). Etiologies, trends, and predictors of 30-day readmission in patients with heart failure. Am J Cardiol.

